# Effect of a Very Low-Calorie Diet on Oxidative Stress, Inflammatory and Metabolomic Profile in Metabolically Healthy and Unhealthy Obese Subjects

**DOI:** 10.3390/antiox13030302

**Published:** 2024-02-29

**Authors:** Neus Bosch-Sierra, Carmen Grau-del Valle, Christian Salom, Begoña Zaragoza-Villena, Laura Perea-Galera, Rosa Falcón-Tapiador, Susana Rovira-Llopis, Carlos Morillas, Daniel Monleón, Celia Bañuls

**Affiliations:** 1 Department of Endocrinology and Nutrition, University Hospital Doctor Peset, Foundation for the Promotion of Health and Biomedical Research in the Valencian Region (FISABIO), 46017 Valencia, Spain; neus.bosch@fisabio.es (N.B.-S.); graudel89@gmail.com (C.G.-d.V.); chsaven@gmail.com (C.S.); begozaragoza1973@gmail.com (B.Z.-V.); laura.perea@fisabio.es (L.P.-G.); rosa.falcon@fisabio.es (R.F.-T.); susana.rovira@uv.es (S.R.-L.); carlos.morillas@uv.es (C.M.); 2 Department of Physiology, INCLIVA Biomedical Research Institute, University of Valencia, 46010 Valencia, Spain; 3 Department of Medicine, University of Valencia, 46010 Valencia, Spain; 4 Department of Pathology, INCLIVA Biomedical Research Institute, University of Valencia, 46010 Valencia, Spain

**Keywords:** obesity, weight loss, metabolic syndrome, oxidative stress, inflammation, metabolome, amino acids, lipid moieties

## Abstract

The purpose of the study was to determine the impact of weight loss through calorie restriction on metabolic profile, and inflammatory and oxidative stress parameters in metabolically healthy (MHO) and unhealthy (MUHO) obese individuals. A total of 74 subjects (34 MHO and 40 MUHO) received two cycles of a very low-calorie diet, alternating with a hypocaloric diet for 24 weeks. Biochemical, oxidative stress, and inflammatory markers, as well as serum metabolomic analysis by nuclear magnetic resonance, were performed at baseline and at the end of the intervention. After the diet, there was an improvement in insulin resistance, as well as a significant decrease in inflammatory parameters, enhancing oxidative damage, mitochondrial membrane potential, glutathione, and antioxidant capacity. This improvement was more significant in the MUHO group. The metabolomic analysis showed a healthier profile in lipoprotein profile. Lipid carbonyls also decrease at the same time as unsaturated fatty acids increase. We also display a small decrease in succinate, glycA, alanine, and BCAAs (valine and isoleucine), and a slight increase in taurine. These findings show that moderate weight reduction leads to an improvement in lipid profile and subfractions and a reduction in oxidative stress and inflammatory markers; these changes are more pronounced in the MUHO population.

## 1. Introduction

Obesity is often associated with an increased risk of metabolic disorders such as type 2 diabetes, hypertension, and dyslipidemia. However, some individuals with obesity do not show signs of these metabolic abnormalities and have a relatively normal metabolic profile, a condition known as metabolically healthy obesity (MHO) [1]. In contrast, those individuals with obesity who exhibit metabolic abnormalities (including impaired glucose metabolism with insulin resistance, hypertension, and atherogenic dyslipidemia) are categorized as metabolically unhealthy or insulin-resistant obese (MUHO) [2]. Some studies suggest that certain factors, such as genetics, distribution of body fat, physical activity, and diet, may contribute to this phenomenon. 

Oxidative stress is one of the mechanisms that has been implicated in the link between obesity and metabolic dysfunction [3]. Oxidative stress occurs when there is an imbalance between the production of reactive oxygen species (ROS) and the capacity of the antioxidant defense system to neutralize them with antioxidants. ROS, including free radicals, are molecules that can damage cells and tissues. In the context of obesity, several factors contribute to increased oxidative stress, such as inflammation, mitochondrial dysfunction, insulin resistance, adipokines, and dyslipidemia [4]. Likewise, ROS-generating systems are involved in several pathophysiological processes characteristic of MUHO, such as inflammation, hypertension, and vascular remodeling (contributing to obesity, type 2 diabetes, and hypercholesterolemia) [5]. Over time, an increased ROS production along with the attenuation of antioxidant enzymes in obese populations may contribute to further complications in insulin-resistant obesity. 

Another key factor implicated in the development of metabolic complications related to obesity is the metabolic response to adipose tissue excess. Metabolomics is the systematic study of the small molecules (metabolites) present in biological systems. It provides a snapshot of the metabolic status and can be used to understand how various biochemical pathways are altered in response to different conditions, including obesity. In this context, metabolomic studies aim to identify and quantify metabolites involved in energy metabolism, lipid metabolism, and other relevant pathways affected by obesity. These changes may be linked to both the primary causes and consequences of obesity. Furthermore, oxidative stress in obesity can directly impact the metabolome [6]. ROS can modify and damage metabolites, influencing their abundance and activity. A glaring example of this is lipid peroxidation or protein carbonyls in obese patients, and their role in atherosclerosis physiopathogenesis [7]. Therefore, metabolomics can provide insights into the inflammatory and oxidative stress status associated with various obesity phenotypes, like differences between MHO and MUHO. 

A very low-calorie diet (VLCD) is a dietary intervention that involves significantly reducing calorie intake to promote rapid weight loss. This type of diet typically provides fewer than 800 calories per day and is often used in the management of obesity. Only a few metabolomics studies have been conducted to investigate the changes in the metabolomic profile following a VLCD in individuals with obesity. VLCD intervention in obese subjects can reduce oxidative stress and inflammation parameters and enhance the antioxidant systems usually reduced in this population [8,9]. Additionally, this improvement in redox imbalance and inflammation levels could be higher in MUHO when compared with MHO [10]. Evidence has demonstrated there is an association between oxidative stress and metabolomics in obesity [11,12]. Furthermore, changes in the metabolomic profile of the obese population correlate with oxidative stress markers, and even some authors have shown that metabolomics can be used to identify metabolic markers of response to a dietetic intervention in the obese population [13,14].

The purpose of the present study was to determine the impact of moderate weight loss through calorie restriction on metabolic profile, inflammatory parameters, and oxidative stress in an obese population categorized as metabolically healthy vs. those with an altered metabolic profile.

## 2. Materials and Methods

### 2.1. Subjects

Participants recruited for the study were individuals seeking treatment for obesity at the Endocrinology and Nutrition Department of University Hospital Dr. Peset in Valencia, Spain. Inclusion criteria encompassed obese individuals aged 18 to 60, maintaining a stable weight (±2 kg) for the prior three months, with a disease duration of at least five years.

Eligible participants had a BMI of ≥30 kg/m^2^, with or without comorbidities. Exclusion criteria included pregnancy, lactation, severe illness, history of cardiovascular or chronic inflammatory disease, and secondary obesity (hypothyroidism, Cushing’s syndrome). The individuals were categorized into two groups following criteria defined by Stefan et al.: MHO and MUHO [1]. MUHO was characterized by the presence of high waist circumference (>102 cm for men and >88 cm for women) and at least two cardiometabolic disorders, including fasting glucose ≥ 100 mg/dL or hemoglobin A1c (HbA1c) ≥ 6.5%, or the use of antidiabetic treatment; systolic blood pressure ≥ 130 mm Hg and/or diastolic blood pressure ≥ 85 mm Hg, or the use of antihypertensive drugs; triglyceride (TG) concentrations ≥ 150 mg/dL; and HDL cholesterol (HDLc) < 40 mg/dL for men and <50 mg/dL for women, or the use of lipid-lowering medication. Conversely, MHO was defined as the absence of all the metabolic syndrome criteria mentioned above except for the presence of high waist circumference.

The study, approved by the Hospital Ethics Committee (Code: 92/18) and aligned with the Declaration of Helsinki, adhered to guidelines from the Spanish Society for the Study of Obesity (SEEDO) [15]. Written consent was obtained from all participants. This study has been registered on ClinicalTrials.gov. The Protocol Registration Identifier is NCT06279780.

### 2.2. Dietary Intervention

After initial evaluation, the subjects underwent two cycles of a very low-calorie diet (VLCD) for 6 weeks each, alternating with a hypocaloric diet (12 weeks).

The dietetic intervention consisted of a VLCD using a liquid formula (Optisource Plus, Nestlé S.A., Vevey, Switzerland), providing 52.8 g protein, 75.0 g carbohydrates, 13.5 g fat, 11.4 g fiber, and essential vitamins and minerals based on Recommended Dietary Allowances (RDA). This formula supplied 2738 kJ/day (654 kcal/day), replacing the participants’ three daily meals. Following this and before the second VLCD cycle, a dietician performed an individualized nutritional assessment to calculate the resting energy expenditure (REE), and personalized hypocaloric diets were prepared, reducing 500 kcal for each individual on their daily caloric expenditure, maintaining the recommended intake of each of the macronutrients (55% carbohydrates, 30% fats, and 15% proteins) for 12-weeks. REE was calculated using validated REE-predictive regression equations, based on the fat-free mass obtained from the impedance analysis instead of body weight.

Dietary monitoring was conducted every 6 weeks with the aim of evaluating the effectiveness of the intervention and meeting the caloric intake objective. This follow-up allowed for the assessment of the adherence through the dietary interview (including dietary intake recording of the last 24 h and frequency of consumption questionnaires, both performed by a trained dietitian) and conducted dietary adaptations if needed. In addition, subjects were clinically monitored by an endocrinologist after starting the first VLCD cycle in order to control symptoms caused by rapid weight loss (such as dizziness, fatigue, and vitamin and mineral deficiency). The clinical and dietary follow-up avoided nutritional biases and uncontrolled variability of the physiological–metabolic response to the dietary factor and also reinforced the follow-up of the diet and the absence of side effects.

Throughout the study, participants received detailed instructions on diet, including portion sizes and cooking methods. Daily consumption of over two liters of calorie-free liquids was recommended, and participants were advised to maintain their regular activity patterns, seeking dietary counseling as needed. Medication prescriptions remained unchanged. Anthropometric parameters (weight, height, BMI, waist circumference, systolic and diastolic blood pressure) and bioelectrical impedance with vector analysis (seca^®^ mBCA 514/515) were measured at baseline and 6 months post-intervention, with blood samples drawn after a 12 h fasting period during both assessments.

### 2.3. Biochemical, Inflammatory and Oxidative Stress Parameters

Blood samples were collected in the morning after overnight fasting to avoid potential confounding influences from circadian cycles. Subjects were assessed for the following parameters: lipid profile, nutritional status, liver and renal function, blood count, coagulation, and hormonal profile. Analytical determinations were carried out in the hospital’s Clinical Analysis Laboratory. 

Proinflammatory cytokine and adipokines levels were assessed using the Luminex^®^ 200 analyzer system (Luminex Corporation, Austin, TX, USA), following the procedure outlined by the MILLIPLEX^®^ Kit manufacturer (Millipore Corporation, Billerica, MA, USA).

Oxidative stress parameters were assessed by a flow cytometry assay (Accuri C6, BD Biosciences, NJ, USA). In brief, an aliquot of whole blood was labeled with anti-CD45 and specific fluorescent probes (Invitrogen, Life Technologies, OR, USA) from leukocytes. We used 2′,7′-Dichlorodihydrofluorescein diacetate (DCFH-DA) to determine total ROS, 5-chloromethylfluorescein diacetate (CMFDA) for glutathione content, dihydroethidium (DHE) for total free radicals and superoxide content, MitoSOX red for mitochondrial ROS production, and tetramethylrhodamine methylester (TMRM) for mitochondrial membrane potential). 

The total antioxidant capacity (TAC) in the serum was measured by the e-BQC portable device (Bioquochem; Oviedo, Spain). The system is based on the measurement of redox potential (charge/period or micro-Coulomb, μC). The samples (100 µL) were dispensed onto a disposable strip. System readings were given for rapid (QA), slow (QB), and total (QT: QA + QB) antioxidant responses. In addition, the levels of SOD and 8-oxo-dG were determined by ELISA, using commercial kits (Cayman Chemical, Ann Arbor, MI, USA).

### 2.4. Metabolomic Analysis

NMR spectra were used to obtain spectra from serum samples from the obese subjects’ cohort. NMR spectroscopy provides the most comprehensive information about a wide range of metabolites, is nondestructive concerning the samples, and requires virtually no sample preparation; thus, it is very suitable for large-scale global metabolic profiling. All samples were analyzed by NMR obtaining a global metabolite profile of up to 40 metabolites including sugars, phospholipids, glycoprotein biomarkers of inflammation, and lipoparticles. NMR spectra were acquired in a Bruker AVANCE III NMR spectrometer (Bruker BioSpin GmbH, Rheinstetten, Germany) operating at a 1H frequency of 600.13 MHz. Trimethylsilylpropanoic acid (TSP) (11202, Deutero, Germany) served as the NMR standard and was added to the serum samples. The metabolomic measurement of serum utilized a 5 mm TXI probe. Each sample consisted of a mixture of 450 µL of serum and 50 µL of phosphate buffer with TSP 2.5 mM and deuterium oxide (D2O) (1.13366, Sigma Aldrich, Hamburg, Germany) for field-locking purposes, transferred into a 5 mm NMR capillary tube (Z172600, Bruker, Rheinstetten, Germany). A single-pulse presaturation experiment, with a 3.95 s acquisition time and 32 transients, was conducted for all samples. All spectra were processed using MestReNova software (MestReNova v14.1.1, Mestrelab Research S.L, Santiago de Compostela, Spain). Corrections were applied to the phase, baseline, and reference of the spectra (to TSP, 0.000 ppm). Normalization to the aliphatic area (0.5–4.5 ppm) was performed. Lipoparticle profiles were generated using in-house scripts and linear regression models calibrated against well-validated, previously published methods [16,17]. Semi-automated MATLAB peak-fitting routines (MATLAB R2014a, MathWorks, Natick, MA, USA) were employed for integrating and quantifying lipid moiety NMR peaks, with final lipid structure levels calculated in arbitrary units as the peak area normalized to the total spectral area. 

### 2.5. Statistical Analysis

Statistical analysis was carried out using SPSS 20.0 (IBM SPSS Statistics, Chicago, IL, USA). Parametric values are presented as mean ± standard deviation (SD), while non-parametric values are reported as median and interquartile range (25th–75th percentile). Qualitative data are expressed as percentages. Bar graphs were generated using the mean ± standard error (SE). Parametric and non-parametric data were compared using the paired Student’s *t*-test and Wilcoxon test, respectively. To examine correlations between variables, a bivariate correlation with Spearman’s Rho was applied. Statistical significance was considered at *p* < 0.05 for all comparisons, with a 95% confidence interval. PLSDA scores, VIPs plots, Circos plots, z-score plots, and correlation matrices plots were created in the R software environment (version 4.1.3). Statistical analysis of these plots was also carried out using R software. To maximize the separation between samples and to identify discriminant patterns, partial least-squares discriminant analysis (PLS-DA) was applied. We adjusted the analysis for age and sex by calculating a linear regression model with these 2 variables for each metabolic feature and using the residues for the PLS-DA analysis. A permutation test was performed to check the overfitting of the PLS-DA models. The multivariate chemometric models were cross-validated with 10-fold Venetian blind cross-validation. In each run, 10% of data were left out of the training and used to test the model. Spectral regions with high variable importance in projections (VIP) coefficients obtained during PLS-DA are more important in providing class separation during analysis, while those with very small VIP coefficients provide little contribution to classification. For the Circos plots, the Circlize and EpiViz plugins were utilized. The statistical significance of the different individual metabolites was also adjusted for age and sex. Finally, all the results were evaluated by cross-validation and the discrimination/prediction power of the different metabolites.

## 3. Results

### 3.1. Body Composition and Biochemical Markers

A total of 74 individuals (69% female), 34 MHO and 40 MUHO, with an age of 41.5 ± 10.4 years and BMI of 41.1 ± 7.5 kg/m^2^ were analyzed. A total of 6% of subjects had type 2 diabetes (of which, only 20% were on oral antidiabetic treatment), 30% had diagnosed hypertension (among which 86% were receiving antihypertensive medication), and 49% had dyslipidemia (of which only 25% were being treated with hypolipemiant treatment). As expected, the MUHO population at baseline showed significantly higher diastolic blood pressure, glucose, TG, Apo B, A1c, insulin, and HOMA-IR, while they had lower HDLc levels than the MHO group (Table 1).

After the intervention, we found a weight loss of 14.3 ± 15.6 kg, with an 18% and 32% fat mass index (FMI) and visceral fat reduction, respectively, plus a significant improvement in resistance without altering reactance. In addition, we observed an improvement in lipid profile (TG, TC, and LDLc), and a significant decrease in both HbA1c and inflammatory parameters (hs-CRP, C3 Protein, and PAI-1). This improvement was more significant in the presence of metabolic syndrome. Furthermore, we found an improvement in adiponectin levels in both groups. In contrast, we did not find an enhancement in the interleukin profile. 

Visceral fat correlated significantly with HDLc levels (r = −0.302, *p* = 0.028), HOMA-IR (r = 0.374, *p* = 0.001), as well as with oxidative damage to DNA/RNA (r = 0.662, *p* < 0.001). We did not find significant correlations between body composition and SOD or with interleukins.

### 3.2. Oxidative Stress Parameters

**Figure 1:**
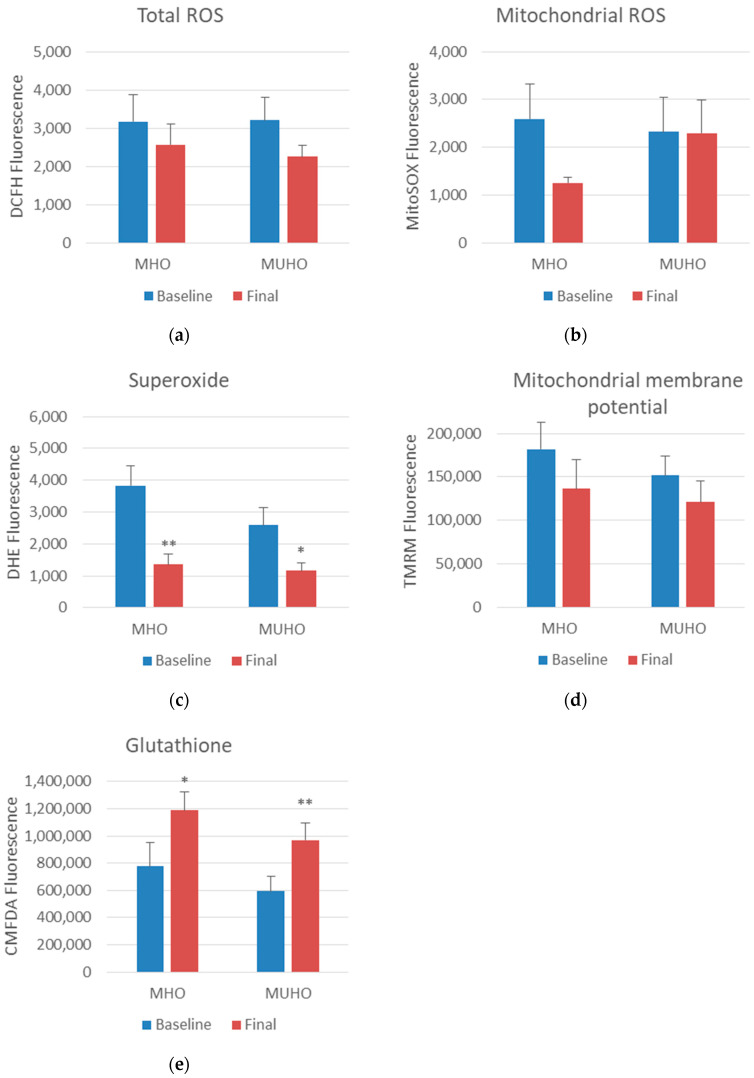
Oxidative stress parameters in metabolically healthy obese (MHO) and metabolically unhealthy obese (MUHO) subjects before and after dietary intervention based on a very low-calorie diet (VLCD). (**a**) Total ROS; (**b**) mitochondrial ROS; (**c**) superoxide; (**d**) mitochondrial membrane potential; (**e**) glutathione. * *p* < 0.05; ** *p* < 0.01 when compared baseline vs. final groups with a paired Student’s *t*-test or U Mann–Whitney (parametric and non-parametric parameters, respectively).

**Figure 2:**
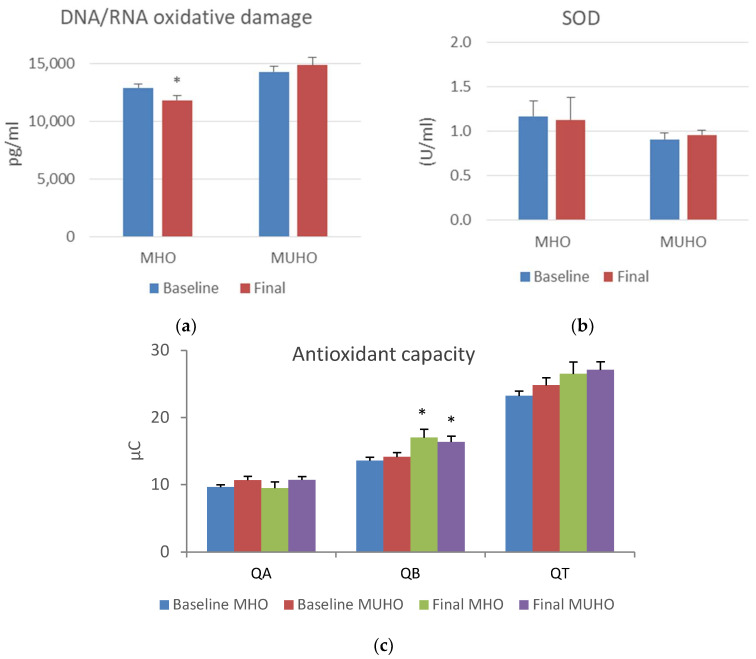
Antioxidant capacity response and oxidative damage in metabolically healthy obese (MHO) and metabolically unhealthy obese (MUHO) subjects before and after dietary intervention based on a very low-calorie diet (VLCD). (**a**) Levels of DNA/RNA oxidative damage measured by 8-OHdG; (**b**) SOD; (**c**) rapid (QA), slow (QB), and total (QT: QA + QB) antioxidant responses. * *p* < 0.05 when comparing baseline vs. final groups with a paired Student’s *t*-test or U Mann–Whitney (parametric and non-parametric parameters, respectively).

Regarding oxidative stress parameters, after the intervention, we observed a significant decrease in superoxide and an increase in glutathione in both groups. Mitochondrial ROS levels showed a decrease only in the MHO group after the diet (*p* = 0.077), although the total ROS and mitochondrial membrane potential did not show significant changes (Figure 1). When we evaluated the antioxidant capacity after the diet, we observed an increase in slow-response antioxidants in both groups, and consequently, a tendency to increase in total antioxidant capacity (*p* = 0.089). In contrast, 8-OHdG levels significantly decreased only in the MHO group. SOD values did not show significant changes (Figure 2).

### 3.3. VLCD-Induced Metabolome Changes

**Figure 3:**
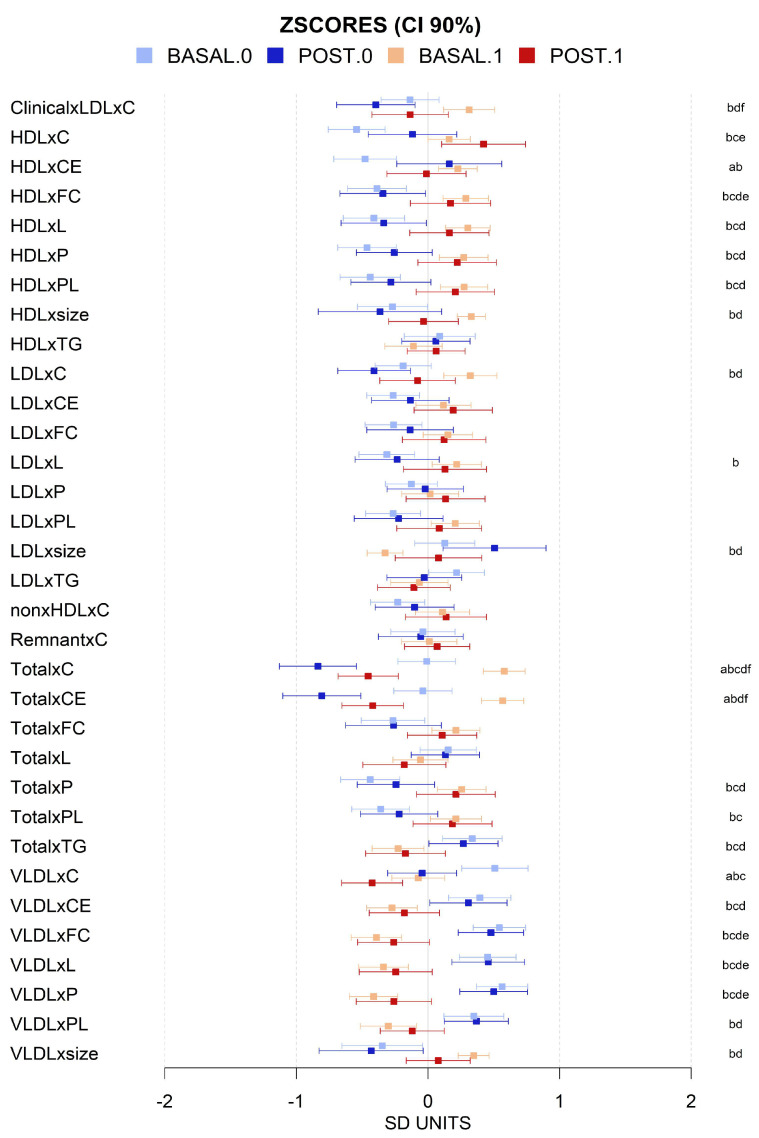
Mean values and 90% CI expressed in SD units for lipoparticles’ general parameters for basal (light colors) and post-intervention (dark colors) levels for individuals with (1, red) and without (0, blue) metabolic syndrome. Multiple pairwise tests with statistical significance at *p* < 0.05 corrected for between-groups comparisons were labeled according to the following code: a, MHO baseline vs. final; b, baseline MUHO vs. MHO; c, baseline MUHO vs. final MUHO; d, final MHO vs. baseline MUHO; e, final MHO vs. MUHO; and f, baseline vs. final MUHO. Key: total cholesterol (C), free cholesterol (FC), cholesteryl esters (CE), triglycerides (TG), phospholipids (PL), total lipids (L), particle size (size), and particle number (P).

**Figure 4:**
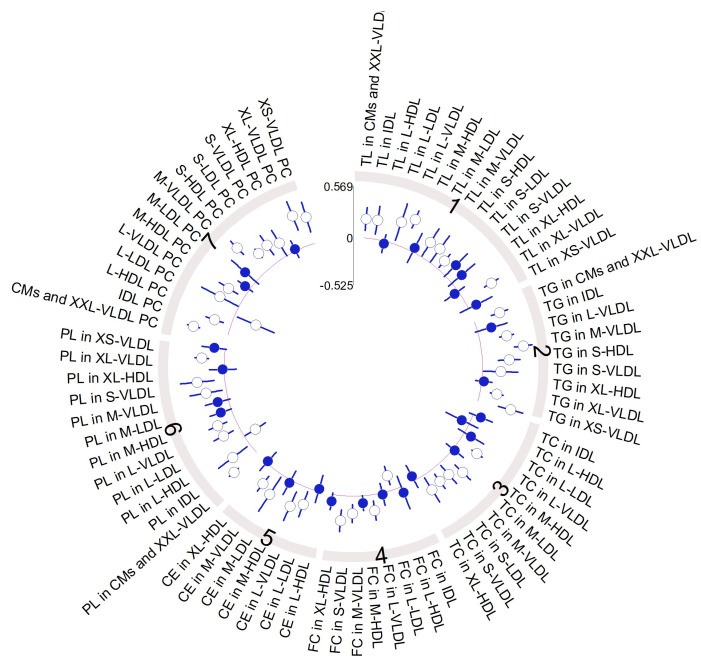
High-resolution profiling of lipoparticles and lipid structures in serum determined by NMR spectroscopy after dietetic intervention. Circos plot representing the mean differences between baseline and post-intervention levels for lipoparticle parameters in standard deviation units (filled and empty circles) and 95% confidence intervals (radial lines around the circle). Values closer to the center of the plot than the red line represent values higher for basal diet than post-intervention, whereas values farther from the center of the plot than the red line represent the opposite trend. Mean significant differences (*p*-value < 0.05) are represented with an empty circle, whereas non-significant mean differences are represented with a filled circle. Key: Same key as in Figure 3 plus XS-, extra small; S-, small; M-, medium; L-, large; XL-, extra large; XXL-, extra extra large; CM, chylomicrons.

**Figure 5:**
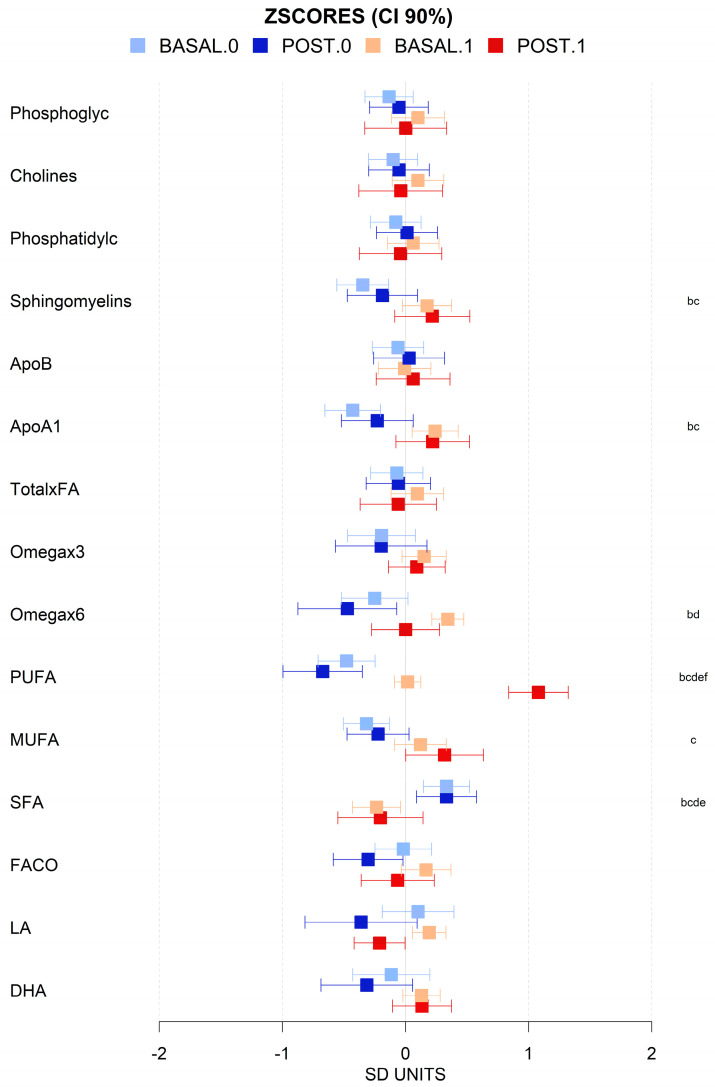
Mean values and 90% CI expressed in SD units for lipid moieties for basal (light colors) and post-intervention (dark colors) levels for individuals with (1, red) and without (0, blue) metabolic syndrome. Multiple pairwise tests with statistical significance at *p* < 0.05 corrected for between-groups comparisons were labeled according to the following code: b, baseline MUHO vs. MHO; c, baseline MUHO vs. final MUHO; d, final MHO vs. baseline MUHO; e, final MHO vs. MUHO; and f, baseline vs. final MUHO. Key: FA, fatty acids; PUFA, total polyunsaturated fatty acids; MUFA, total monounsaturated fatty acids; SFA, total saturated fatty acids; FACO, total carbonyl groups in fatty acids; LA, linoleic acid; DHA, docosahexaenoic acid.

**Figure 6:**
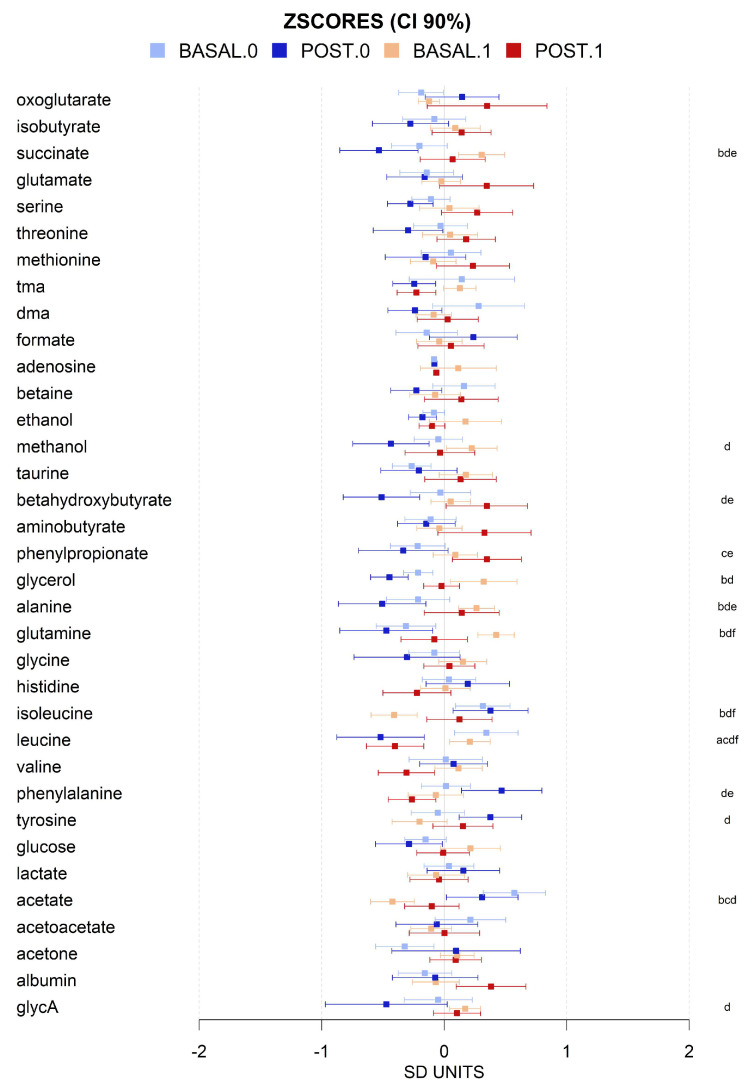
Mean values and 90% CI expressed in SD units for non-lipidic for basal (light colors) and post-intervention (dark colors) levels for individuals with (1, red) and without (0, blue) metabolic syndrome. Multiple pairwise tests with statistical significance at *p* < 0.05 corrected for between-groups comparisons were labeled according to the following code: a, MHO baseline vs. final; b, baseline MUHO vs. MHO; c, baseline MUHO vs. final MUHO; d, final MHO vs. baseline MUHO; e, final MHO vs. MUHO; and f, baseline vs. final MUHO. Key: tma, trimethylamines; dma, dimethylamines; GlycA, acetyl groups in glycoproteins.

There was a wide diversity in the metabolomic profiles of the cohort and differences, both before and after intervention, which were relatively subtle when the data were normalized. However, some differences between MHO and MUHO metabolomic profiles were detected before the intervention. At the lipoparticle level, the ratios of total cholesterol (C), cholesteryl esters (CE), triglycerides (TG), phospholipids (PC), and total lipids (TL) were, in general, higher in MUHO than in MHO, except for VLDL particles. MUHO showed larger VLDL particle size and smaller LDL size, which after the intervention showed a trend to be closer to MHO, with smaller VLDL and larger LDL. This was a general trend for all the metabolomic parameters analyzed with MUHO parameters which were closer to those of MHO after the intervention. We also observed that the intervention shifts, in general, the global lipoprotein profile towards more cholesterol and cholesterol esters in HDL than in VLDL, and more TGs in VLDL and less in LDL (Figure 3 and Figure 4). The intervention induced changes in the high-resolution lipoparticle profile. Figure 4, adjusted for age and sex, shows a higher impact also in these subparticle classes (LDL and VLDL) with increased ratios of lipid species in small LDL particles and smaller ratios in HDL and VLDL. The analysis of lipid species showed that, in general, neither the basal status nor the intervention was associated with large significant changes in them (Figure 5). The basal levels of sphingomyelins and omega 6 lipids seem to be higher in MUHO than in MHO before the intervention. Interestingly, the same trend for closer ApoA1 values between MHO and MUHO after than before the intervention was observed. The most dramatic effect of the intervention and the most notable difference is the high increase in total PUFAs in MUHO after the intervention. The intervention seems to also decrease omega 6, carbonyl in fatty acids, and linoleic acid levels in both MUHO and MHO, but the effect was not significant. At the level of small and general non-lipidic metabolites (Figure 6), we did not observe significant differences between MUHO and MHO profiles before the intervention. Some trends were noticed, but only the differences in succinate, acetate, and glutamine were significant. Different from the lipidic parameters, the intervention had a parallel effect on most of the metabolites with a general decrease, for example, in glycerol, alanine, succinate, formate, leucine, valine, and glycoprotein A (GlycA), and a slight increase in tyrosine and taurine (Figure 6). Interestingly, some metabolites showed a larger separation between MHO and MUHO after the intervention, showing an opposite trend to that observed in lipidic parameters. Among them, the changes in phenylalanine and beta-hydroxybutyrate were significant. 

**Figure 7:**
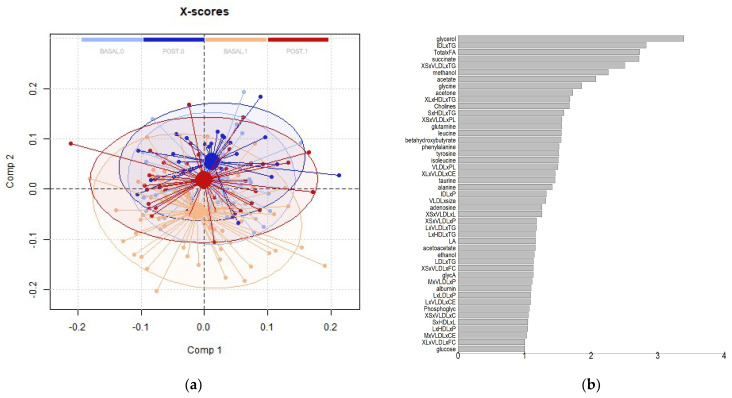
PLS-DA model for discrimination between basal (light colors) and post-intervention (dark colors) levels for MUHO (1, red) and MHO (0, blue) individuals. All models were built using 2 latent variables (LV). The score plot (**a**) shows a 95% confidence ellipse in the same colors and suggests that MUHO after intervention is a metabolome closer to MHO individuals. VIP scores (**b**) show the most relevant metabolites in this model. Cross-validation parameters: RMSECV 0.534, R2CV: 0.642; ROC Curve AUC: 0.76.

To further explore the potential relationships between MUHO and MHO and the impact of the intervention, we performed a PLS-DA model for discrimination between baseline and final intervention levels for MUHO and MHO individuals (Figure 7). PLSDA allows for the handling of high-dimensional datasets, making it effective for simultaneously analyzing numerous metabolites. Its robustness in dealing with correlated variables ensures accurate identification of patterns and discrimination between different groups. The method’s capacity for dimensionality reduction is crucial for visualizing and interpreting complex metabolomic data. PLSDA accommodates small sample sizes common to our study, providing reliable results in scenarios where traditional methods may falter. The scores plot showed that, as observed for most of the lipidic parameters and some non-lipidic species, MUHO after intervention is a metabolome closer to MHO individuals. The impact of the intervention at the global metabolome levels follows the same directions (upwards in component 2) for both MHO and MUHO, with a larger impact on MUHO individuals. VIPs scores showed that the most relevant lipid species were triglycerides in lipoparticles of different sizes and phospholipids in VLDL lipoparticles and, at the general metabolites level, glycerol, succinate, acetate, glycine, acetone, cholines, glutamine, leucine, phenylalanine, and beta-hydroxybutyrate (Figure 7).

**Figure 8:**
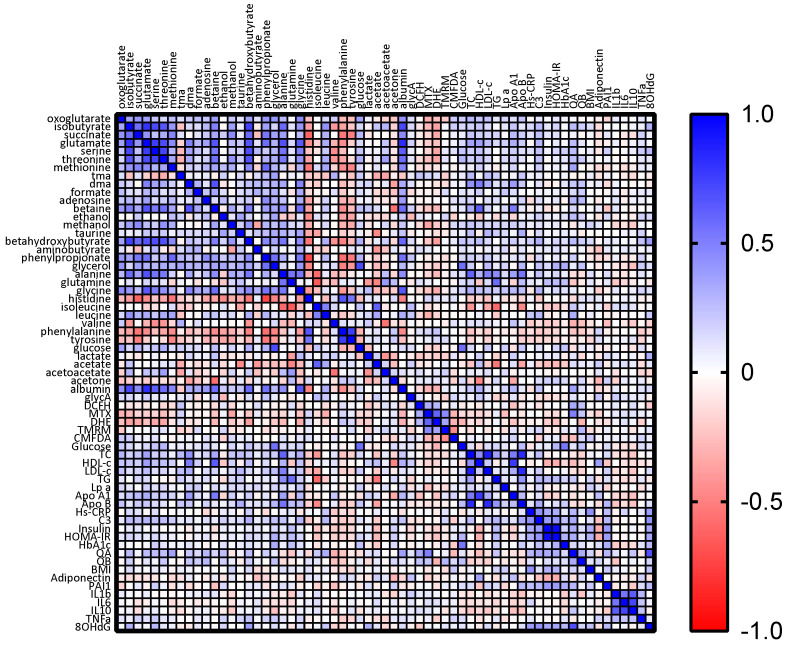
Correlation matrices between metabolomic and biochemical parameters. Stronger correlations are represented by darker colors. Negative correlations are represented in red whereas positive correlations are represented in blue.

Spearman correlation coefficients between baseline metabolite levels and clinical and oxidative stress parameters are shown in Figure 8. It is noteworthy to mention that leucine was negatively correlated with mitochondrial ROS, superoxide levels, and IL-1b. We also found a positive correlation between leucine levels and both hs-CRP and C3 proteins. Conversely, isoleucine was positively correlated with SOD levels, and in turn negatively with superoxide levels and HOMA-IR. Valine was positively correlated with glutathione levels. Taurine was positively correlated with BMI and C3 protein, but not with glutathione or other oxidative stress parameters. Succinate was positively correlated with C3 protein, and negatively correlated with superoxide levels. Finally, GlycA was negatively correlated with both glutathione and IL-1b levels.

## 4. Discussion

This interventional study assessed the impact of moderate weight loss through a calorie-restricted diet on oxidative stress and inflammatory profile and ran an extensive biomarker profiling of 176 lipids, metabolites, and proteins across two distinct obese subgroups, MHO and MUHO, an unexplored field as of yet. In our adult obese population, the moderate weight loss achieved by adherence to a two 6-week-VLCD cycle interspersed with a hypocaloric diet for 12 weeks improved both anthropometric and biochemical parameters, and ameliorated the inflammatory response, being more substantial in the MUHO population. In addition, total superoxide was reduced and glutathione and slow-response antioxidant levels increased. Thus, the weight loss through the dietetic intervention contributed to a reduction in oxidative stress. This response was associated with an improvement in insulin resistance coupled with a decrease in acute-phase proteins like hs-CRP, C3 protein, and PAI-1.

Oxidative stress has been identified as a key mechanism in insulin resistance and obesity pathogenesis [5]. The excess of adipose tissue, and subsequent low-grade chronic systemic inflammation, facilitates ROS production induced by the innate immune system activation. Furthermore, the excess of adipose tissue has been recognized as a source of pro-inflammatory cytokines such as TNF-α, IL-1, and IL-6. As a result, pro-inflammatory cytokines produce an increase in ROS production and lipid peroxidation; in turn, ROS production will induce the further release of pro-inflammatory cytokines, adhesion molecules, and growth factors expression, coupled with a disrupted adipokine production (like adiponectin reduction) [4,18]. In addition, the free fatty acid overload due to obesity can cause cellular dysfunction, especially in the mitochondria. Mitochondria dysfunction is considered a key factor in insulin resistance development. As adipose tissue increases, mitochondrial transcription levels in adipocytes decrease, culminating in impaired glucose utilization in this tissue [4]. Both mitochondrial dysfunction and ROS overproduction are associated with glucose impairment, pro-inflammatory agents, and lipid peroxidation. Thus, both play a critical role in the pathogenesis of comorbidities associated with obesity.

Our data show that weight loss through dietetic intervention with VLCD enhances redox status in the obese population, regardless of being MHO or MUHO. This was accompanied by an improvement in inflammatory response and adiponectin, although we did not find significant differences in pro-inflammatory cytokines levels. Caloric restriction has been shown to improve consequential indicators of oxidative stress through pro-inflammatory cytokines in obese populations [19,20]. Regarding intervention with VLCD, our results concur with López-Domènech et al., where a dietetic intervention with VLCD in an obese population showed an improvement in superoxide production and antioxidant systems [8]. Furthermore, if we assess redox status according to the presence of MUHO or MHO, Bañuls et al. found that the MUHO population presented a significant deterioration in oxidative stress parameters such as total and mitochondrial ROS production, and mitochondrial membrane potential, compared to MHO individuals [21]. We did not find a significant amelioration in pro-inflammatory cytokines levels or SOD activity after weight loss through VLCD, unlike other authors [8,9,22]. However, different studies presented similar results as ours, despite an improvement in inflammatory parameters like hs-CRP; Oberhauser et al. and Asghari et al. did not observe a significant decrease in pro-inflammatory cytokines after VLCD in the obese population [23,24]. Still, these contradictory results can be explained by different studied individuals (most trials did not differentiate between MHO vs. MUHO). Also, there are considerable differences in the duration of the intervention and the dietetic intervention per se (VLCD followed with or without a low-calorie diet, and the use of enteral formulas vs. conventional diet). 

In relation to DNA oxidative damage, it can be considered as an indirect parameter of an imbalance in redox status. Chronic inflammation is associated with obesity and is strongly involved in DNA oxidative lesions. Increases in pro-inflammatory cytokine levels due to adipose tissue excess in obesity can induce DNA damage through ROS-increased levels [25]. Quantifying DNA damage could be potentially useful in the early risk assessment and prevention of obesity-associated comorbidities. We observed a significant depletion in 8-OHdG levels only in the MHO group. This result is in line with the significant reduction in 8-OHdG levels found in the obese population after weight loss through VLCD and low-calorie diet by Ozvald et al. and Heilbronn et al. [26,27]. Although altered 8-OHdG levels in both obesity and diabetes are described in the literature, there are no studies that analyze the differences in DNA oxidative damage levels between MHO and MUHO. Moreover, Di Minno et al. found in their meta-regression analyses that 8-OHdG levels were increased by the presence of the male gender, whilst no association was found with the presence of diabetes or BMI [28]. This could explain why we do not see the same result in MUHO vs. MHO, given that in our studied population the majority are women.

Metabolomics can identify metabolic abnormalities associated with metabolic syndrome early, even associated with cardiovascular disease [11,29,30]. Additionally, some authors have found that metabolites can be used to predict future weight gain, independently from anthropometric, lifestyle, and glycemic factors [31]. Basal metabolomic profiles between MHO and MUHO are very intermixed, and very few statistically significant differences can be detected except for some lipid species and the content of triglycerides and cholesteryl esters in lipoparticles. However, we observed that our intervention had a notable effect on lipidic species with a reduction in total cholesterol and cholesteryl esters in both MHO and MUHO and a total redistribution of lipid species and lipoparticles both at the content and size levels towards larger but unchanged in number LDL particles and more HDL particles. In general, the intervention closes the metabolomic gap between MHO and MUHO, with a few exceptions regarding small metabolites like phenylalanine and beta-hydroxybutyrate. The most significant effect of the dietary intervention at the lipid parameters level is a reduction in the small LDL parameters and an increase in average LDL particle size. Small LDL has been linked to cardiovascular risk and a reduction is likely to be linked to the beneficial effects of the intervention [32]. 

Interestingly, after the dietetic intervention, we found a reduction in lipid carbonyl levels, along with an increased ratio of polyunsaturated fatty acids, with a potentially beneficial effect. Carbonyl groups are functional groups consisting of a carbon atom double-bonded to an oxygen atom. In long-chain fatty acids, they are crucial in beta-oxidation and ATP production. They have also been linked to oxidative stress. We have not found studies that evaluate the level of carbonyls after weight loss either in human or animal models. There was only one study that evaluated the effect of a dietetic intervention on lipid carbonyl levels in a rat model of non-alcoholic fatty liver disease after a high-fat diet [17]. It is logical to assume that a VLCD will reduce total fat intake, which could be reflected in reduced levels of both unsaturated and saturated fatty acids. There are very few studies that evaluate the effect of VLCD on unsaturated fatty acid levels since most address the effect of unsaturated fatty acid supplementation added to caloric restriction. Our finding is in line with the few studies that found a significant reduction in unsaturated fatty acid levels (both monounsaturated and polyunsaturated fatty acids) after a caloric restriction [31]. However, the PUFA ratio was significantly improved after the intervention, similarly to the findings by Mills et al. after a lifestyle intervention in obese pregnant women [33]. This may be caused by the use of a polyunsaturated fatty acid-enriched liquid formula instead of a conventional very low-calorie diet without meal substitutes. Positively, the unsaturated fatty acid level improvement could be associated with the inflammatory response enhancement observed in our population.

Succinate has been considered a respiratory substrate of the mitochondrial electron transport chain, but recently other physiological functions have been attributed to it. Succinate excessive level is regarded as a marker of hypoxia and tissue damage, and also a pro-inflammatory signal that promotes immune activation. Succinate levels are strongly increased in inflammatory-related pathologies, including obesity and type 2 diabetes, and are associated with an increased risk of cardiovascular disease [34]. In our population, we found significantly increased levels of succinate in MUHO vs. MHO. Despite other studies reflecting an association between circulating succinate levels and both BMI and HOMA-IR [35], we did not find any studies that compare differences between MHO and MUHO. After our intervention, we observed a significant reduction in succinate levels in both groups. Astiarraga et al. also observed that weight loss and metabolic improvement promoted by bariatric surgery in obese patients caused a reduction in fasting levels of succinate [36]. 

Concerning amino acid metabolism, we appreciated a decrease in leucine and valine (both BCAAs), and in alanine, both in MHO and MUHO, after the weight loss. There is great evidence that BCAA levels are associated with insulin resistance, and tend to be increased in obesity [37]. Several cohort studies with long-term follow-up state that BCAA levels could be predictive of insulin sensitivity deterioration and type 2 diabetes development [38]. Nonetheless, the mechanism of action of BCAA in insulin resistance is still unclear. Two non-mutually exclusive possibilities are hypothesized. BCAA plasma level could increase because of decreased insulin sensitivity, which would affect BCAA metabolism. Likewise, BCAA or some of their derivates could promote insulin resistance through inhibiting insulin-induced AKT phosphorylation; also, it has been hypothesized that the mammalian target of rapamycin complex 1 (mTORC1) could be overactivated by excessive levels of BCAA, leading to a reduction in insulin-stimulated glucose uptake caused by insulin receptor substrate (IRS) degradation and reduced Akt-AS160 activity [38,39]. Weight loss could improve BCAA levels, and therefore could contribute to improving insulin sensitivity, but results in the scientific literature are contradictory. Zhang et al. found that baseline BCAA levels were associated with the improvement in HOMA-IR in overweight patients after a very low-protein and -calorie diet [40]. Hernández-Alonso et al. observed a significant reduction in leucine and valine levels in overweight and obese patients after a low-glycemic index diet [41]. In contrast, after weight loss through VLCD in obese patients, Sayda et al. did not find a significant reduction in BCAA plasmatic levels [42]. In consequence, our findings regarding BCAA levels after weight loss are in line with some authors; however, these results cannot be easily compared since there are huge differences in sample population characteristics, type, and duration of the dietetic intervention. 

Our results also showed a small increase in taurine levels after the dietetic intervention. Several human studies have demonstrated the beneficial role of taurine in obesity. There is evidence showing that increased taurine consumption could ameliorate obesity and its related metabolic disorders [43]. This protective effect would be mediated by taurine regulation of adipose tissue mass, modulation of lipid metabolism, its involvement in the correct functioning of antioxidant enzymes, and its implication in the proper functioning of the β-pancreatic cell [43,44]. There are few studies that evaluate taurine levels in obese populations without using supplementation. Rosa et al. found similarly that obese women had lower taurine levels in comparison with lean women [45]. 

Regarding alanine, it has also been established that the obese population presents higher alanine levels [31]. Guash-Ferré et al. found that baseline alanine levels in obese patients were significantly higher in those with type 2 diabetes [46]. Given that alanine is a highly gluconeogenic amino acid, it could be hypothesized that an increased amount of alanine released by the visceral adipose tissue to systematic circulation would contribute to hyperinsulinemia and insulin resistance development [31]. We observed a significant decrease in alanine levels in MHO and MUHO groups after the intervention, similarly to Geidenstam et al. after a diet-induced weight loss in the obese population [47]. Despite this, we did not observe significant correlations with HOMA-IR. 

In relation to GlycA, it is considered a novel marker of low-grade inflammation that reflects the glycosylation of acute-phase proteins. It has been proposed to reflect the systemic acute phase response better than hs-CRP, and it is well established that GlycA is associated with pathologies that cause metabolic alterations, like diabetes and obesity [48]. We found a significant reduction in glycA levels in both groups after the weight loss. This result is in line with the reduction observed by Collins et al. after diet in overweight patients [48]. Even further, Huffman et al. displayed GlycA level reduction after caloric restriction in healthy participants [49].

Understanding the intricate relationship between oxidative stress and metabolomics in obesity is crucial, in order to unravel potential biomarkers for obesity and related complications and develop targeted therapeutic approaches. However, there are few studies that evaluate oxidative stress and metabolomics association; in addition, they are usually focused on indirect oxidative stress parameters, such as pro-inflammatory mediators, and not on direct parameters like ROS or antioxidant content. There is a clear association between BCAA levels (or their derivates) and oxidative stress promotion. This mechanism would be mediated by an increased ROS production through NADPH oxidase and Akt-mTOR activation. An excess of BCAA concentration can also induce in vitro NF-kB production, but on the other hand, it can also induce the expression of defense proteins against oxidation and inflammation with the transcription factor Nrf2 [50,51]. Concerning taurine, it is clear that its supplementation in obese animal models can improve oxidative stress response [52,53]. Additionally, some authors have found an association between taurine supplementation and adipose tissue function in both animals and humans [54,55]. This would be mediated by the critical role taurine plays in the synthesis of proteins involved in the respiratory chain, regulating the activity of enzymes that neutralize ROS, glutathione activation, and inactivating ROS itself [56]. Rosa et al. observed a significant hs-CRP improvement after taurine supplementation in obese women [45]. Similarly, Haidari et al. showed a significant hs-CRP reduction in obese women after taurine supplementation combined with a hypocaloric diet [57]. Additionally, Samadpour et al. found that taurine supplementation along with exercise had a synergetic effect on decreasing HOMA-IR levels [58]. Our results show an association between BCAA and oxidative stress response in the obese adult population. Nevertheless, although taurine is also associated with indirect oxidative stress parameters like hs-CRP, we did not find significant associations with ROS or glutathione levels. Apart from amino acids, succinate levels also showed an association with the oxidative stress response. There is evidence that accumulated succinate levels during inflammatory macrophage activation would enhance mitochondrial ROS production [59]. Lastly, GlycA was also related to glutathione and IL-1b levels. There is evidence that GlycA (reflection of glycans) and oxidative stress are closely related, but the mechanism of action is not still clear [60]. 

We would like to comment on certain limitations of the study. First, because the study was conducted on an obese population classified as MHO or MUHO profile, caution should be given when generalizing other populations. Second, while NMR spectroscopy has the capability to quantitatively capture a comprehensive metabolomic pattern for blood, the complexity of samples and metabolite concentrations may lead to overlapped signals, despite the application of validation methods. In addition, because not all the amino acid derivates were reported, we cannot discard that there could be other not found correlations between amino acid metabolites and oxidative stress. Third, despite using a liquid formula product in the VLCD phase, during the conventional hypocaloric diet there was greater variability in adherence. Fourth, we did not take into account other factors that can modulate oxidative stress, such as exercise. The strengths of this study included an interventional study design and relatively long-term follow-up, a large sample size, and well-characterized metabolic phenotypes of obese populations of both MHO and MUHO subjects (since most of the studies focus on diabetes or insulin-resistant populations). One of the most noteworthy findings from our study is the metabolomics analysis, adjusted by age and sex, in order to explore a possible association between oxidative stress and metabolomic biomarkers after weight loss through dietetic intervention. However, it is essential to implement strategies in usual clinical practice that guarantee long-term follow-up of these populations, since the observed results will depend on their adherence to the achieved dietary changes and weight loss.

## 5. Conclusions

Weight loss by calorie restriction in obese subjects contributes to a lower cardiovascular risk associated with an improvement in lipid profile and insulin resistance, and a reduction in oxidative stress and inflammatory markers, these changes being more pronounced in MUHO. Metabolomic profile analysis by NMR unveiled not only quantitative changes in fat but also qualitative shifts in overall lipid composition. Additionally, we observed distinctions in the unsaturation and carbonyl content of these lipids as well as changes in BCAAs.

Metabolomic analysis and inflammatory profiling are valuable tools for assessing the effects of weight loss. These analyses can help healthcare professionals better understand how weight loss affects metabolism and inflammation and may be useful in monitoring the metabolic health of individuals with obesity. Therefore, additional research is needed to achieve a more thorough comprehension of the underlying mechanisms.

## Figures and Tables

**Table 1:** Anthropometric, metabolic, and inflammatory parameters in metabolically healthy obese (MHO) and metabolically unhealthy obese (MUHO) subjects before and after dietetic treatment based on a very low-calorie diet (VLCD).

	MHO		MUHO	
	Baseline	Final	Baseline	Final
N (females %)	34 (76.4)		40 (62.5)	
Weight (kg)	112.1 ± 23.5	99.3 ± 21.7 ***	115.9 ± 20.7	102.9 ± 19.6 ***
BMI (kg/m^2^)	39.0 (35.5; 44.6)	33.9 (30.7; 39.8) ***	40.5 (36.9; 43.0)	36.3 (30.7; 39.6) ***
Waist (cm)	117.0 ± 14.9	105.5 ± 15.8 ***	121.9 ± 15.8	108.7 ± 16.0 ***
Fat mass index (kg/m^2^)	18.9 (15.8; 23.7)	15.3 (12.3; 19.6) ***	19.0 (16.8; 21.4)	16.1 (12.5; 18.9) ***
Free fat mass index (kg/m^2^)	21.0 ± 3.5	19.9 ± 3.1 ***	21.0 ± 2.5	20.2 ± 2.4 ***
Visceral fat (L)	4.1 (3.0; 6.2)	2.8 (1.7; 4.4) ***	4.9 (3.7; 8.5)	3.1 (2.0; 4.8) ***
Resistance (Ω)	526.5 ± 81.1	552.3 ± 87.7 ***	526.5 ± 66.1	546.2 ± 71.8 ***
Reactance (Ω)	50.0 ± 9.8	51.1 ± 9.3	50.7 ± 7.5	51.1 ± 9.0
Phase angle (°)	5.4 ± 0.5	5.3 ± 0.5 *	5.5 ± 0.6	5.3 ± 0.6 **
SBP (mmHg)	128 ± 10	119 ± 13 **	132 ± 15	120 ± 14 ***
DBP (mmHg)	78 ± 10	76 ± 9	83 ± 10 #	76 ± 9 ***
Glucose (mg/dL)	92.3 ± 10.0	90.5 ± 9.3	110.5 ± 35.6 ##	98.3 ± 12.6 *
Insulin (μU/mL)	17.8 ± 10.7	12.5 ± 6.0 ***	26.5 ± 17.6 #	15.6 ± 8.9 ***
HOMA-IR	3.5 (2.1; 6.0)	4.1 (3.6; 5.3) *	5.6 (4.4; 8.1) ###	4.8 (4.1; 5.7) **
A1c (%)	5.4 (5.1; 5.7)	5.3 (5.0; 5.6) *	5.6 (5.4; 6.0) #	5.4 (5.2; 5.6) ***
TC (mg/dL)	182.4 ± 39.6	173.8 ± 41.7 *	192.2 ± 33.5	177.5 ± 39.4 **
HDLc (mg/dL)	49.5 (41.8; 56.0)	46.0 (39.8; 55.3)	41.0 ± (36.3; 49.8) ##	41.0 ± 11.7
LDLc (mg/dL)	113.7 ± 31.5	108.4 ± 33.4 *	114.8 ± 26.6	111.4 ± 35.3
TG (mg/dL)	96.5 (76.5; 106.5)	79.5 (65.2; 96.5)	162.5 (127.0; 222.5) ###	113.0 (80.3; 156.5) ***
Apo A1 (mg/dL)	141.0 (131.3; 164.0)	128.0 (118.0; 149.0) *	136.5 (123.0; 158.5)	123.5 (111.5; 142.8) **
Apo B (mg/dL)	94.3 ± 23.0	86.8 ± 25.4 **	108.0 ± 24.5 #	93.1 ± 30.6 **
Lp(a) (mg/dL)	10.0 (4.8; 28.5)	11.5 (6.0; 29.0)	8.5 (3.0; 29.8)	11.0 (5.0; 27.0)
Uric acid (mg/dL)	5.5 (5.0; 6.0)	4.8 (4.2; 5.6) *	5.7 (4.9; 6.8)	5.2 (4.7; 6.3) *
hs-CRP (mg/L)	8.2 (4.3; 16.9)	7.0 (2.6; 13.2) **	8.8 (4.6; 13.9)	5.0 (3.1; 11.3) *
C3 Protein (mg/dL)	137.4 ± 23.4	124.5 ± 17.8 ***	143.6 ± 17.4	131.9 ± 17.5 ***
Adiponectin (μg/mL)	23,495.5 ± 14,729.1	31,306.1 ± 27,093.7 **	21,001.1 ± 12,412.5	24,674.9 ±14,965.5 **
PAI-1 (ng/mL)	114.3 (77.2; 133.2)	89.1 (71.4; 122.7) *	116.4 (100.3; 177.2)	107.8 (77.6; 136.7) ***
IL-1b (pg/mL)	1.5 (0.9; 2.4)	1.4 (0.8; 2.6)	1.3 (0.8; 2.3)	1.3 (0.8; 2.4)
IL-6 (pg/mL)	0.6 (0.2; 4.5)	0.3 (0.2; 6.6)	0.3 (0.2; 1.0)	0.2 (0.2; 1.3)
IL-10 (pg/mL)	12.3 (2.0; 32.9)	14.6 (3.0; 52.5)	9.7 (2.1; 20.4)	9.8 (2.3; 18.3)
TNF-α (pg/mL)	11.0 (9.5; 14.4)	10.9 (8.7; 14.8)	12.4 (11.3; 15.0)	12.7 (11.1; 15.4)

Data are presented as mean ± SD for parametric data and median (IQ range) for non-parametric parameters. * *p* < 0.05; ** *p* < 0.01; *** *p* < 0.001 when compared with a paired Student’s *t*-test or U Mann–Whitney (parametric and non-parametric parameters, respectively). # *p* < 0.05; ## *p* < 0.01; ### *p* < 0.001 when compared with an independent Student’s *t*-test or Wilcoxon test. BMI: body mass index; SBP: systolic blood pressure; DBP: diastolic blood pressure; HOMA-IR: insulin resistance index; A1c: hemoglobin A1c; TC: total cholesterol; HDLc: high-density lipoprotein cholesterol; LDLc: low-density lipoprotein cholesterol; TG: triglycerides; Apo A1: apolipoprotein A1; Apo B: apolipoprotein B; hs-CRP: high-sensitivity C-reactive protein; PAI-1: plasminogen activator inhibitor-1; IL: interleukin; TNF-α: tumoral nuclear factor alpha.

## Data Availability

The data presented in this study are available on request from the corresponding authors. The data are not publicly available due to ethical reasons.
